# A user-centred implementation strategy for tuberculosis contact investigation in Uganda: protocol for a stepped-wedge, cluster-randomised trial

**DOI:** 10.1186/s12889-023-16510-0

**Published:** 2023-08-17

**Authors:** Achilles Katamba, Amanda J Gupta, Patricia Turimumahoro, Emmanuel Ochom, Joseph M Ggita, Suzan Nakasendwa, Leah Nanziri, Johnson Musinguzi, Rachel Hennein, Moorine Sekadde, Colleen Hanrahan, Raymond Byaruhanga, Erez Yoeli, Stavia Turyahabwe, Adithya Cattamanchi, David W Dowdy, Jessica E Haberer, Mari Armstrong-Hough, Noah Kiwanuka, J. Lucian Davis

**Affiliations:** 1 Uganda Tuberculosis Implementation Research Consortium, Kampala, Uganda; 2 Makerere University School of Medicine, Kampala, Uganda; 3 Yale School of Public Health, New Haven, CT USA; 4 Johns Hopkins Bloomberg School of Public Health, Baltimore, MD USA; 5 Yale School of Medicine, New Haven, CT USA; 6 National TB and Leprosy Programme, Ministry of Health, Kampala, Uganda; 7 Massachusetts Institute of Technology, Cambridge, MA USA; 8 University of California Irvine, Irvine, CA USA; 9 Massachusetts General Hospital, Boston, MA USA; 10 Harvard University, Cambridge, MA USA; 11 School of Global Public Health, New York University, New York, NY USA; 12 Makerere University School of Public Health, Kampala, Uganda

**Keywords:** Tuberculosis, Stepped-wedge trial, Contact investigation, Uganda, Implementation science

## Abstract

**Background:**

Tuberculosis(TB) is among the leading causes of infectious death worldwide. Contact investigation is an evidence-based, World Health Organisation-endorsed intervention for timely TB diagnosis, treatment, and prevention but has not been widely and effectively implemented.

**Methods:**

We are conducting a stepped-wedge, cluster-randomised, hybrid Type III implementation-effectiveness trial comparing a user-centred to a standard strategy for implementing TB contact investigation in 12 healthcare facilities in Uganda. The user-centred strategy consists of several client-focused components including (1) a TB-education booklet, (2) a contact-identification algorithm, (3) an instructional sputum-collection video, and (4) a community-health-rider service to transport clients, CHWs, and sputum samples, along with several healthcare-worker-focused components, including (1) collaborative improvement meetings, (2) regular audit-and-feedback reports, and (3) a digital group-chat application designed to develop a community of practice. Sites will cross-over from the standard to the user-centred strategy in six, eight-week transition steps following a randomly determined site-pairing scheme and timeline. The primary implementation outcome is the proportion of symptomatic close contacts completing TB evaluation within 60 days of TB treatment initiation by the index person with TB. The primary clinical effectiveness outcomes are the proportion of contacts diagnosed with and initiating active TB disease treatment and the proportion initiating TB preventative therapy within 60 days. We will assess outcomes from routine source documents using intention-to-treat analyses. We will also conduct nested mixed-methods studies of implementation fidelity and context and perform cost-effectiveness and impact modelling. The Makerere School of Public Health IRB(#554), the Uganda National Council for Science and Technology(#HS1720ES), and the Yale Institutional Review Board(#2000023199) approved the study and waived informed consent for the main trial implementation-effectiveness outcomes. We will submit results for publication in peer-reviewed journals and disseminate findings to local policymakers and representatives of affected communities.

**Discussion:**

This pragmatic, quasi-experimental implementation trial will inform efforts to find and prevent undiagnosed persons with TB in high-burden settings using contact investigation. It will also help assess the suitability of human-centred design and communities of practice for tailoring implementation strategies and sustaining evidence-based interventions in low-and-middle-income countries.

**Trial registration:**

The trial was registered(ClinicalTrials.gov Identifier NCT05640648) on 16 November 2022, after the trial launch on 7 March 2022.

**Supplementary Information:**

The online version contains supplementary material available at 10.1186/s12889-023-16510-0.

## Background

Over 4 million people with active tuberculosis (TB) disease worldwide went undiagnosed in 2021 [1], making community-based active TB case finding and prevention critical for reducing TB transmission and incidence [2]. Contact investigation is a World Health Organization (WHO)-endorsed intervention for screening, diagnosis, treatment, and prevention of TB among individuals reporting close contact with persons newly diagnosed with active TB [3]. Systematic reviews and meta-analyses show that contact investigation identifies active TB disease in about 2–5% of close contacts and latent TB infection in another 30–50%, with variations in yield attributable to design-related bias and heterogeneous screening and testing algorithms and implementation strategies [4–6]. Based on this large body of evidence, WHO first endorsed contact investigation in low- and middle-income countries in 2012 and updated its recommendations in 2021. Real-world data on how to best implement TB contact investigation is needed [7–9].

Several comparative effectiveness trials have contributed to these efforts. An individually randomised, controlled trial in Brazil found that intensified TB screening among household contacts (including as-needed home visits and follow-up evaluation and treatment of latent and active TB in clinics) decreased TB incidence by 15% over five years relative to usual care without contact investigation [10]. The ZAMSTAR study, a cluster-randomised trial in 24 communities in Zambia and South Africa, found that household contact investigation with TB preventive therapy (TPT) for eligible contacts decreased TB transmission by 55% and active TB prevalence by 18% relative to clinic-based enhanced case-finding plus TPT, although the differences were not statistically significant [11]. A cluster-randomised trial in 70 districts in Vietnam demonstrated that inviting household TB contacts for clinic-based TB symptom and chest x-ray screening every six months for two years significantly increased the cumulative incidence of TB diagnoses and treatments by 2.5-fold compared with usual care without contact investigation [12].

However, in a comparative meta-analysis of 15 contact investigation implementation projects in programmatic settings in high-burden countries, most involving community health workers (CHWs) and performance incentives, the yield of newly diagnosed persons with TB among all contacts varied widely, from 0.1 to 6.2% [13]. Lower screening uptake by people with TB and close contacts was associated with lower diagnostic yield. Several recent pragmatic cluster-randomised trials in sub-Saharan Africa aimed to improve implementation using novel, multi-component facilitation strategies, including home sputum collection with HIV testing by CHWs [14–16] or household members [17], text messaging to deliver test results and follow-up instructions [14], and client-targeted financial incentives to attend clinics for screening [15]. Unfortunately, none of these strategies improved TB evaluation, case finding, or mortality relative to passively screening contacts [9]. Further advances in implementation are needed.

One novel approach for developing implementation strategies is human-centred design [18], also known as design thinking [19] or user-centred design. [20] Drawing on diverse influences, including consumer product design [18], social innovation [21], and human-computer interaction engineering [20], human-centred design is a creative approach that employs qualitative methods, rapid prototyping, and iterative field-testing. [22] Its goal is to understand problems from the end user’s perspective and identify unexpected solutions they will embrace. [23] We recently employed these participatory methods with healthcare workers, clients, and community members in Uganda, to co-create a novel user-centred implementation strategy for TB contact investigation.

Because sustaining complex interventions like contact investigation is a challenge in high-burden, resource-constrained settings [9], we included implementers and clients among our end users. Our specific strategy was to promote the creation of communities of practice among implementers. Communities of practice, groups of practitioners with a shared domain who come together for mutual support and exchange tacit knowledge, were first described among West African apprentices who reported that learning most often occurred through interactions with each other rather than with the masters [24, 25]. Communities of practice are often touted in the health and management sectors, but data about if and how they improve service delivery and health outcomes are lacking [24, 26], as are details about strategies to foster their development [27]. We recently demonstrated the core components required to establish a community of practice among CHWs implementing TB contact investigation in Uganda [28, 29].

 We are currently conducting a stepped-wedge, cluster-randomised trial with nested mixed-methods studies to evaluate whether a user-centred strategy targeting clients and implementers can improve and sustain delivery of TB contact investigation in Uganda. Our study has three aims: (1) to determine the implementation, effectiveness, and public health impact of TB contact investigation delivered via a user-centred compared to a standard implementation strategy; (2) to identify processes and contextual factors that influence the implementation, effectiveness, and public health impact of the user-centred implementation strategy; and (3) to compare the costs and epidemiological impact of the user-centred and standard implementation strategies. The user-centred strategy has two parts: (a) four client-centred facilitation tools delivered under the brand name *Tuli Wamu Nawe* (a Luganda phrase meaning “We are Together with You”); and (b) healthcare-worker-centred tools designed to foster the formation of a CHW community of practice. The goal is to improve and sustain standard TB contact investigation delivery following Uganda National TB and Leprosy Programme guidelines. Our primary hypothesis is that the user-centred strategy will result in more close contacts completing TB evaluation and being diagnosed and treated for TB than the standard strategy. We further hypothesise that the user-centred strategy will (1) be more feasible, acceptable, and appropriate for clients and CHWs than the standard strategy, (2) increase self-efficacy and perceived social support among CHWs, and (3) be cost-effective and capable of reducing national TB incidence over a 10-year horizon relative to the standard strategy.

## Methods/design

### Trial design

**Fig. 1:**
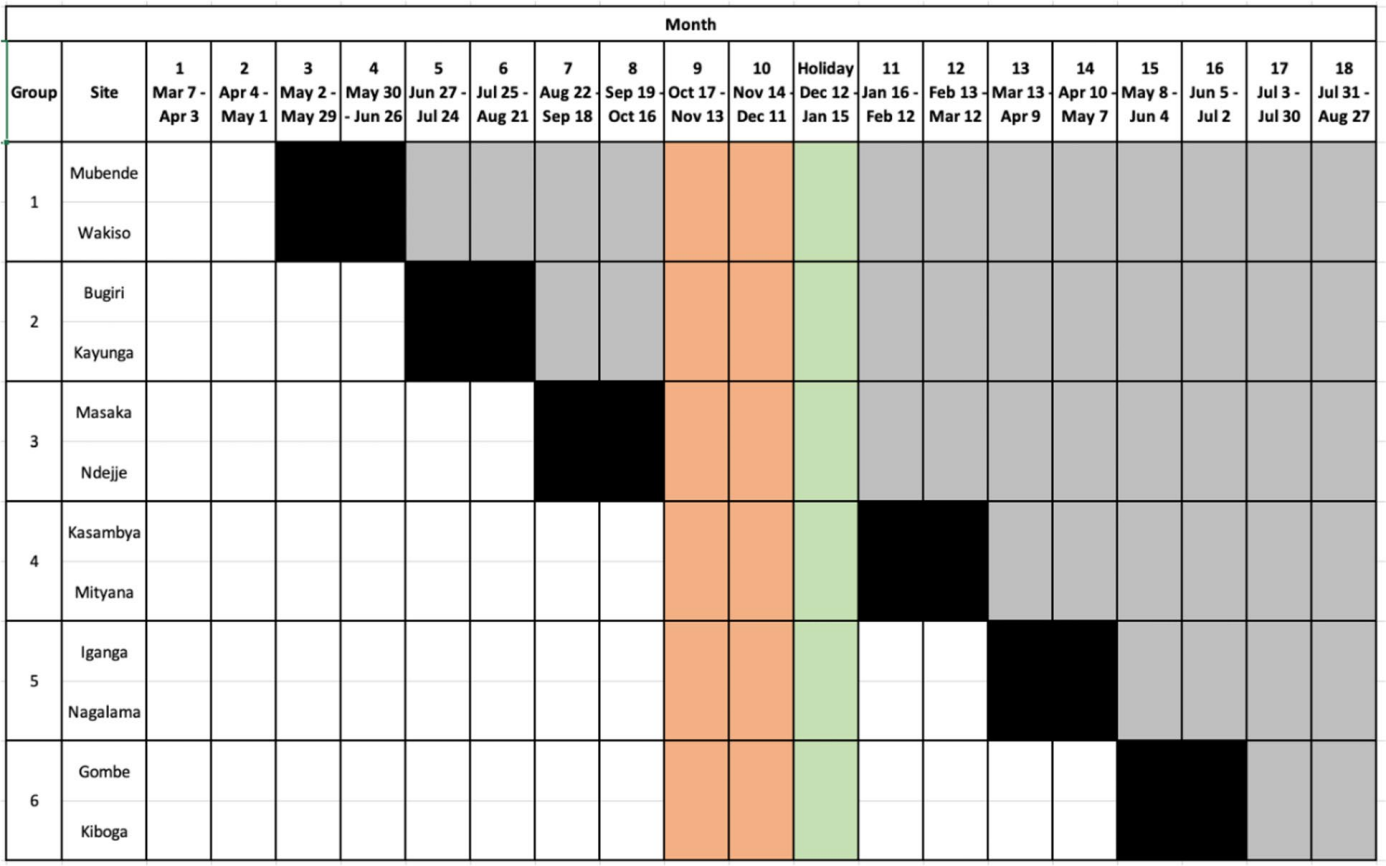
Study schema, with post-randomisation site and cluster assignments, for the trial in which new enrolments began on 7 March 2022, and are scheduled to end on 27 August 2023. The schema also shows unplanned study enrolment interruptions due to Ebola Virus Disease lockdowns between 18 October 2022, and 11 December 2022, and planned enrolment interruptions due to low anticipated enrolment during the annual holiday period between 12 December 2022 and 15 January 2023

 We are conducting a complete (closed) stepped-wedge, cluster-randomised Type III hybrid implementation-effectiveness trial to determine if a user-centred delivery strategy can improve the proportion of close contacts of people with TB completing TB evaluation [30]. The trial scheme includes six eight-week transition steps following a randomly determined order of cluster switching from pre-intervention to intervention phase (Fig. 1) to achieve a 1:1 ratio of periods allocated to the standard and user-centred implementation strategy exposure periods after excluding the transition periods as a buffer. The trial began on 7 March 2022 and included an unplanned, 14-week extension of the transition phase between Steps 3 and 4 due to an outbreak of Ebola Virus Disease in Uganda between September 2022 and January 2023. The trial steering committee recommended excluding from analysis all data collected from new clients enrolled between 18 October 2022 and 15 January 2023 after a public health order restricting all public movement around two sites in two districts interrupted training and enrolment. This study protocol follows the SPIRIT (Standard Protocol Items: Recommendations for Interventional Trials) reporting guidelines and includes a completed SPIRIT checklist in the Supplemental materials.

### Study setting

Uganda is one of thirty WHO-designated high HIV-TB burden countries, with an estimated annual TB incidence of 199 per 100,000 persons and treatment coverage of only 82% [1]. All TB management units in Uganda offer free facility-based TB evaluation and treatment through the Uganda Ministry of Health following National TB Programme Guidelines [31]. In 2019, Uganda issued additional guidelines endorsing contact investigation as a routine service after several years of demonstration projects in Kampala and selected districts [32]. We chose 12 healthcare facilities with a high volume of TB case notifications located outside Kampala in Central Uganda to provide a mixture of rural and peri-urban facilities (Table 1). We coordinated the final site selection with the National TB Programme to ensure alignment with public health priorities.

**Table 1:** Healthcare facilities participating in the trial

Study Site	Classification	Volume of Contacts
Bugiri District Hospital	Peri-Urban	Low
Gombe District Hospital	Rural	Low
Iganga District Hospital	Peri-Urban	High
Kasambya Health Centre IV	Rural	Low
Kayunga Hospital	Rural	High
Kiboga District Hospital	Rural	High
Masaka Regional Referral Hospital	Urban	High
Mityana Hospital	Rural	High
Mubende Regional Referral Hospital	Peri-Urban	High
Nagalama Hospital	Rural	Low
Ndejje Health Centre IV	Peri-Urban	Low
Wakiso Health Centre IV	Peri-Urban	Low

### Eligibility criteria

To select sites for trial inclusion, we reviewed National TB Programme data for all TB units to identify healthcare facilities (1) reporting ≥ 12 new persons with TB per month and (2) located outside but within 180 km of Kampala District. Research staff conducted site visits to verify eligibility by auditing on-site TB treatment registers and to determine the willingness of facility leaders to participate in the trial. We excluded facilities whose administrators declined to participate.

To select individuals for inclusion as persons with TB, we are recruiting (1) adults or children recorded as having TB disease in the on-site national TB treatment register, following national contact investigation guidelines [32], if they also (2) reside ≤ 40 km from the enrolling healthcare facility. We are excluding persons who (1) lack the capacity to consent to contact investigation, (2) report no close contacts, (3) have possible or confirmed drug-resistant TB, (4) have undergone TB contact investigation within the previous two months, or (5) decline to refer their close contacts for TB contact investigation.

To select individuals for inclusion as close contacts, we are recruiting adults or children reporting ≥ 12 cumulative hours with the person with TB inside an enclosed space within the previous three months, as per national contact investigation guidelines [32]. We are excluding close contacts who (1) lack the capacity to consent to contact investigation, (2) are currently taking treatment for active TB, or (3) decline to participate in contact investigation.

For the Aim 2 fidelity and context studies, we are directly observing collaborative improvement meetings and conducting demographic and social-behavioural surveys, in-depth interviews, and focus-group discussions with all CHWs delivering the standard and user-centred implementation strategies. Research staff are also purposively sampling 20 persons with TB and 50 close contacts by age, gender, and HIV status for in-depth interviews during the trial intervention periods. For the Aim 3 time-and-motion and costing studies at selected study sites, we are including CHWs and healthcare workers who are 1) ≥ 18 years of age, 2) employed by the study site, and 3) involved in conducting or supervising TB contact investigation at the healthcare facility.

### Standard TB contact investigation procedures

According to Uganda national guidelines [32], healthcare workers should invite all persons newly diagnosed with TB with ≥ 1 close contact to participate in contact investigation. CHWs should then visit those who agree to participate at home or another acceptable location to enumerate all eligible close contacts. CHWs should screen consenting contacts for active TB disease and arrange for microbiologic, clinical, and/or radiographic evaluation of anyone reporting TB symptoms. CHWs should collect sputum from symptomatic close contacts aged ≥ 5 years, transport it to healthcare facility laboratories for microbiologic evaluation, and report test results back to contacts; CHWs should refer those < 5 years or unable to expectorate sputum to a healthcare facility for evaluation. Finally, they should refer adolescents and adults with unknown HIV status (i.e., not known to be living with HIV and not tested within the prior three months) and all contacts with TB symptoms to healthcare facilities for HIV testing.

### Implementation strategies

#### Standard implementation strategy for TB contact investigation

The standard implementation strategy for contact investigation in Uganda is for national TB programme instructors to deliver on-site training on TB contact investigation procedures to TB unit staff, including CHWs, followed by supportive supervision from the National TB Programme district supervisor and on-site focal person during implementation. To prepare for the trial, we ensured that each site had 3–4 CHWs to serve all persons starting TB treatment at that site and provided a monthly salary and travel allowance for site visits at standard local rates. We collaborated with the National TB Programme to deliver the standard training on the national TB contact investigation guidelines to all CHWs and TB unit staff. We also trained all CHWs to accurately record all contact investigation data in the programmatic registers and in electronic case-record forms (CommCare, Dimagi, Boston, MA) using trial-issued electronic tablets. The electronic data collection system includes decision support to prompt CHWs to deliver all contact investigation services [14]. Research staff securely send weekly reports on missing data to CHWs.

#### User-centred implementation strategy for TB contact investigation

The user-centred strategy for implementing contact investigation adds four client-facing and three healthcare-worker-facing components to the standard implementation strategy components of training, supportive supervision, and electronic data collection. We describe these seven components of the user-centred strategy below, including how and why each was selected.

Previous studies show that a lack of TB knowledge, anticipated and enacted stigma, cost and travel obstacles, and dissatisfaction with public health services are the main barriers that prevent people with TB and their close contacts from fully participating in contact investigation [33, 34]. To address these barriers, we collaborated with a professional design team from IDEO.org, a non-profit organisation specialising in human-centred design. Small, rotating teams of 2–3 designers from North America and East Africa led the study team through four design phases. First, we undertook a five-week “inspiration” phase involving direct observation of contact investigation procedures and interviews and focus groups with end-users and other community members, often facilitated by games, group art projects, and role plays. We sorted important ideas and themes using adhesive notes on an open wall in the project offices. Second, we embarked on a six-week “ideation and rough prototyping” phase, involving brainstorming potential solutions including “sacrificial concepts” intended to elicit client and healthcare worker feedback and help designers develop implementation interventions and synthesise prototype services. Third, the Uganda study team conducted a “iteration and live prototyping” phase to pilot and collect feedback on the implementation components, extended from six to 12 months because of COVID pandemic interruptions. Finally, the design team refined all implementation and training materials during a three-week design sprint.

**Fig. 2:**
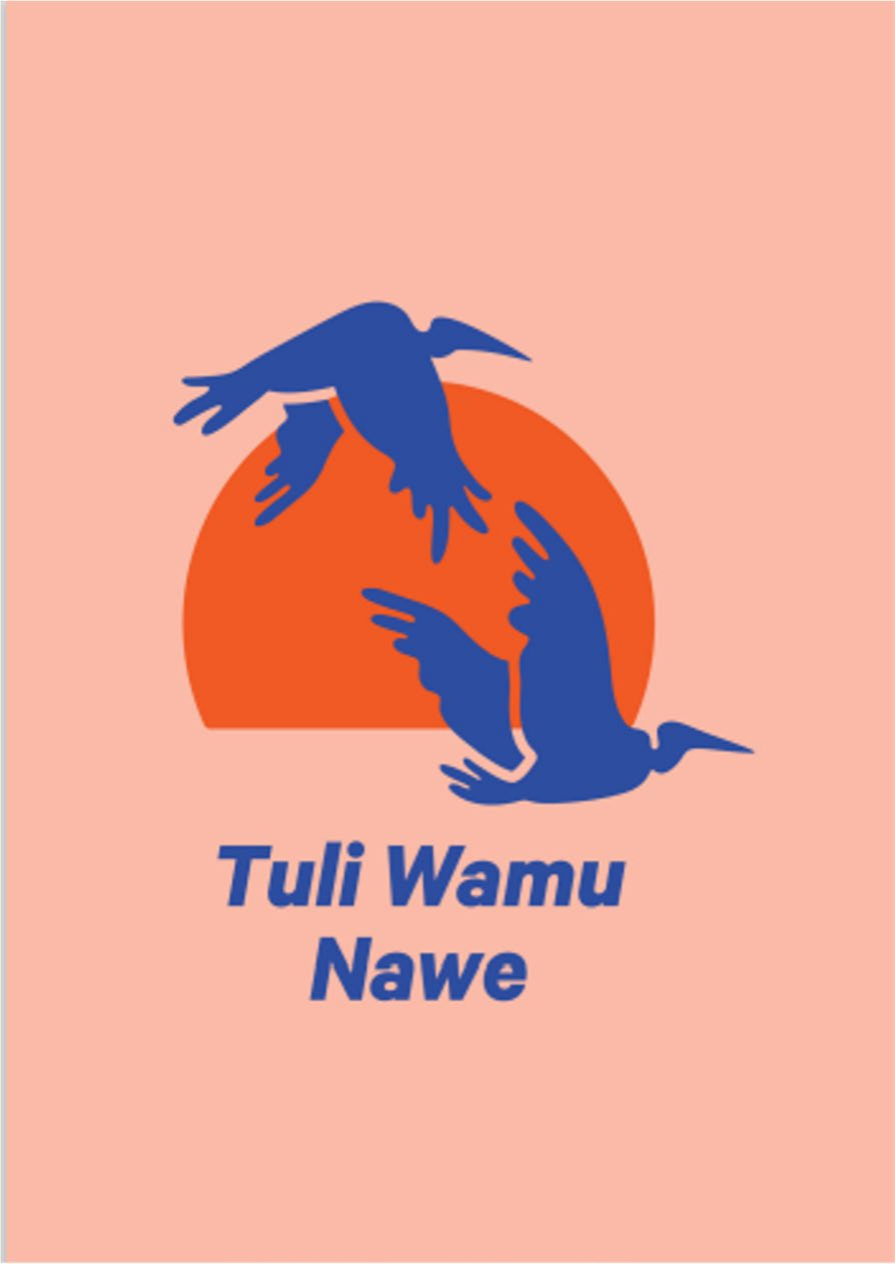
The *Tuli Wamu Nawe* (translation “We are together with you”) brand logo appears on the uniforms of CHWs and CHRs and on other programme materials. It is designed to communicate the user-centred values of the programme and help clients recognise and trust different members of the contact investigation team when interacting in different settings

 The final client-facing components of the user-centred strategy consist of four implementation facilitation tools delivered as the branded “Tuli Wamu Nawe” package. These tools include (1) a TB education booklet, (2) a contact identification algorithm, (3) an instructional video on sputum collection, and (4) a community health rider (CHR) service staffed by commercial motorcycle taxi drivers who transport clients, CHWs, and sputum samples at no cost to users. Finally, to help clients recognise and trust different members of the contact investigation team, CHWs and CHRs carry identification cards and uniforms (i.e., shirts, rain ponchos, and umbrellas). These are imprinted with a small Tuli Wamu Nawe graphic logo depicting two Marabou storks, a beloved local bird species in Uganda, flying in tandem in front of a sunrise (Fig. 2).

### TB education booklet

CHWs provide all persons with TB with a TB education booklet, which includes basic information about TB disease in an engaging visual format to address knowledge barriers and help persons with TB disclose their diagnosis to contacts. This need and solution were identified through the human-centred design process. The booklet is available in Luganda or English and covers how TB spreads, how contact investigation can benefit participants, and how people diagnosed with TB can prepare for contact identification by considering all venues where they might have interacted with close contacts.

### Contact identification algorithm

Embedded within the electronic case record forms at cross-over, the contact identification algorithm helps enumerate close contacts more accurately by systematically exploring where persons with TB spent time with others (e.g., home or other residences, work, religious and social gatherings) before diagnosis. During the HCD process, designers observed and heard from CHWs that contact enumeration was a particular challenge. The algorithm aims to identify more close contacts per household and do so earlier in the contact investigation process to facilitate efficient planning of community visits and to improve the public health yield, a particular need given the high costs of contact investigation [35].

### Sputum collection video

Formatted for display to clients on provider tablets or phones, the sputum collection video provides standardised instructions and encouragement to help contacts overcome the anticipated stigma associated with sputum expectoration [36] and learn how to expectorate high-quality sputum samples. Developed by InTuneForLife, a non-profit organisation of media professionals, the video has been shown to increase the yield of TB diagnoses in Pakistan [37]. InTuneForLife later worked with local designers and voice actors to culturally adapt the video’s images, sounds, and voices for Luganda [38] and English [39] for use in Uganda.

### Community health riders

Community Health Riders (CHRs) are commercial motorcycle-taxi drivers paid to transport CHWs, persons with TB, and close contacts to and from the community at no charge. Developed during the human-centred design process, the CHR concept adapts and expands the “hub-rider” model used to transport TB specimens between healthcare facilities to overcome the barriers CHWs face in finding and reaching households and linking contacts to clinics [14, 34]. Healthcare workers at study sites suggest motorcycle drivers with exceptional knowledge of the people and geography of local communities to become CHRs. The CHRs are trained to assist CHWs by displaying the sputum collection video to clients on CHRs’ smartphones and transporting sputum samples to laboratories. CHRs are also trained to interact professionally with clients, including adhering to privacy protections, using personal protective equipment appropriately, handling and transporting medical specimens properly, and following road safety measures.

The three healthcare-worker-facing components of the user-centred contact investigation strategy were selected as activities previously shown helpful in fostering a CHW community of practice for implementing contact investigation [29]. These components include (1) collaborative improvement meetings, (2) audit and feedback reports, and (3) a local digital chat-application group (i.e., WhatsApp).

### Collaborative improvement meetings

Collaborative improvement meetings take place weekly, rotating through several formats. These include internal meetings of each site’s CHWs and CHRs, virtual meetings (e.g., Google Meet) with external CHWs and CHRs at a paired implementation site, and in-person meetings of CHWs and CHRs with other healthcare workers at their own site. Each type of meeting seeks to review implementation performance, identify challenges, and brainstorm solutions. CHWs may also organise structured didactics on best TB care practices led by TB focal persons and other clinicians.

### Audit and feedback reports

Starting during cross-over, an unblinded data manager prepares weekly individual-level and monthly facility-level reports on CHW and CHR performance at each step of TB contact investigation. These reports are electronically delivered to a secure, password-protected server (Box Inc, Redwood City, CA, USA) on CHW tablets for group review during collaborative improvement meetings. Drawing on evidence from behavioural economics about promoting cooperation in groups [40], the reports seek to inform CHWs and CHRs about their performance, establish performance norms, and motivate improvement through shared accountability and identification of role models.

### Digital chat groups

At cross-over, we will connect all CHWs and CHRs at each health facility and their supervisor, the local TB focal person, on a local WhatsApp chat group. The goal is to facilitate coordination of client care, exchange of technical assistance, shared accountability, and social support outside the collaborative improvement meetings. The schedule of procedures for the standard and user-centred strategies is summarised in Table 2.

**Table 2:** Participant schedule of procedures

Activity	Participant	Implementation Strategy	Location	Duration
Assessing for eligibility	Person with TB	Both	Facility/Telephone	3 min
Invitation to contact investigation	Person with TB	Both	Facility/Telephone	3 min
Intake interview	Person with TB	Both	Facility/Telephone	10 min
1. Education & counselling				
a. Routine TB education	Person with TB	Standard	Facility/Telephone	20 min
b. TB education booklet	Person with TB	User-centred	Facility/Telephone	15 min
2. Contact enumeration				
a. Standard contact enumeration	Person with TB	Standard	Facility/Telephone	5 min
b. Contact person identification algorithm	Person with TB	User-centred	Facility/Telephone	10 min
3. Assessing for eligibility	Contact person	Both	Community	3 min
4. Invitation to contact investigation	Contact person	Both	Community	3 min
5. Symptom screening	Contact person	Both	Community	15 min
6. Instruction on sputum collection				
a. Standard sputum instruction^a^	Contact person	Standard	Community	6 min
b. Sputum collection video^a^	Contact person	User-centred	Community	3 min
7. Sputum collection^a^	Contact person	Both	Community	10 min
8. Linkage to care				
a. Travel to facility^b^	Contact person	Standard	Community/Facility	~ 10–60 min
b. Transport to facility by CHR^b^	Contact person	User-centred	Community/Facility	~ 10–60 min
9. Evaluation by clinician^b^	Contact person	Both	Facility	~ 45 min

*Abbreviations: TB* Tuberculosis

^a^Only for those needing TB evaluation. ^b^Only for those unable to produce sputum during community visits or those with indeterminate TB evaluation results

### Training on the user-centred implementation strategy

We selected three expert CHWs from non-trial sites to deliver the trainings at the start of each eight-week transition period. CHWs were chosen for their excellent communication skills and prior experience with the user-centred implementation strategy during the live prototyping phase of the human-centred design work. Before the first transition period, research staff led a training of trainers for the expert CHWs requiring 24 contact hours over one month. Each transition period begins with a three-day in-service training in an off-site classroom setting near the healthcare facility led by the regional and district TB supervisors for that site, two research staff, and one expert CHW. All CHWs (3–4 per site), all CHRs (2 per site, plus 2 back-ups), TB unit staff, and at least one representative each from the laboratory, the HIV unit, and the facility administration are required to attend. The curriculum includes process mapping, interactive didactic sessions, engaging printed materials, short videos, and role plays on contact investigation and the user-centred strategy. On days 4–10, the expert CHWs move to the site with the trainees for peer-mentored co-enrolment, including supportive supervision and daily debriefing sessions to streamline processes and agree on key learnings. Research staff are also on-site for support. CHWs and CHRs graduate to independent enrolment after they have been observed going through the entire process of user-centred delivery of contact investigation and have performed satisfactorily.

After the initial 10-day training, CHWs and CHRs proceed with independent enrolment while the expert CHWs and research staff move to train the second site for that transition period. After two weeks, the trainers return to debrief with the CHWs and CHRs and introduce the concept of communities of practice. Trainers participate in the subsequent collaborative improvement meetings to address operational challenges, provide mentoring on directing discussion, and observe trainee progress. Mentoring continues in-person and remotely until the end of the transition period or when the training team decides the trainees are ready for independent implementation and all training activities and site visits end.

### Assignment of interventions

We hosted a public randomisation ceremony in Kampala, Uganda, on 11 February 2022. We used sequential random drawing to randomly allocate the order in which participating healthcare facilities would transition to the user-centred strategy (Fig. 1) and to stratify the randomisation by the average monthly volume of persons with TB. To stratify, we randomly assigned healthcare facilities to one of six groups, each including one higher- and one lower-volume facility. A National TB Programme representative first drew a labelled tag from an opaque bag to determine whether higher- or lower-volume site representatives would choose. Then, one clinician from each of the six sites drew one of six tags (labelled for the six possible partner sites) from an opaque bag without replacement. Next, we randomly assigned each facility pair to a cross-over time by asking a senior National TB Programme representative to draw six numbered balls from an opaque bag without replacement. The first number drawn was used to assign the initiation time for Group 1, the second for Group 2, and so on, until all six groups had been assigned a place in the sequence.

### Blinding

Blinding the intervention strategy is not feasible given the stepped-wedge, cluster-randomised trial design because the strategies are implemented at the health facility level. All health facilities receive the standard strategy followed by the user-centred strategy. CHWs cannot be blinded to outcomes because they are responsible for collecting all process and outcome data. All investigators will be blinded to all aggregated analyses of TB outcomes by study period and will only be unblinded upon trial completion.

### Criteria for discontinuing or modifying the implementation strategy

Because the user-centred implementation strategy is a minimal-risk behavioural intervention that poses no greater danger to clients than routine care, we do not anticipate a need to discontinue or modify it. However, we will monitor the safety of travelling with CHRs and report any accident-related injury to the IRBs as an adverse event, graded for severity and relatedness to the trial. No concomitant care is prohibited during the trial. Any contact diagnosed with TB while undergoing contact investigation will have free access to treatment at the participating healthcare facilities.

### Outcomes

Our primary outcome is a measure of implementation effectiveness, the proportion of symptomatic close contacts who complete TB evaluation within 60 days of the TB treatment initiation date for the index person with TB. We also have several secondary outcomes. First, to measure clinical effectiveness (also known as yield), we will determine the proportion of contacts diagnosed with active TB and initiated on active TB treatment and the proportion of contacts initiating TB preventative therapy (TPT) within 60 days of the index person with TB’s treatment initiation date. Second, to measure public health effectiveness, we will determine the counts of contacts diagnosed with active TB and of those initiating TPT. Third, as measures of public health efficiency and timeliness, we will measure the proportion of all persons diagnosed with TB who are contacts and the time to completion of contact evaluation. We will compare these outcomes during the standard and user-centred implementation periods and determine the between-period differences, testing the null hypothesis that there is no difference between periods. Finally, the hybrid implementation-effectiveness outcomes structure draws on the RE-AIM Evaluation Framework [41], and additional analyses will assess adoption of contact investigation and reach to index persons and contacts within the contact investigation cascade [42].

### Data collection and management

**Fig. 3:**
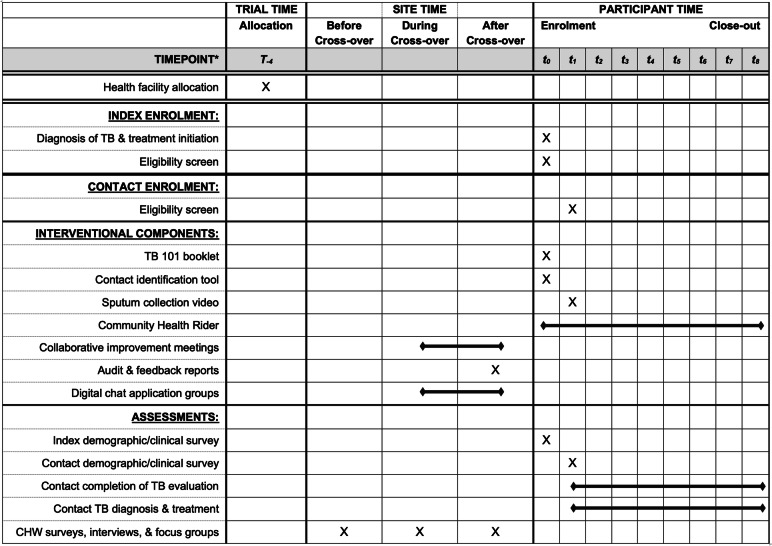
SPIRIT flow diagram showing enrolment, interventional, and assessment procedures. TB, tuberculosis. T, time relative to start of the trial. t, time relative to enrolment of the index person living with TB. *Time indicated in subscripts, measured in weeks relative to the start of the stepped-wedge implementation trial (T), to enrolment of the index person living with TB (t), or to each site’s time of cross-over.

 A SPIRIT flow diagram for the trial is provided in Fig. 3. CHWs will collect individual participant outcomes for Aim 1 by directly reviewing facility TB registers, including the presumptive TB register, lab register, TB treatment register, and TPT register. CHWs will single-enter all data electronically on password-protected, wirelessly connected Android tablets into a customised CommCare application (Dimagi, Boston, MA). Data entry can occur online or offline, and data automatically syncs to a HIPAA-certified, password-protected, encrypted server whenever connectivity is detected. The application forces responses to all questions and requires correction of out-of-range entries as a condition to proceed. The data manager will review datasets weekly for missing and impossible values. We will provide CHWs with regular data management reports throughout the trial, including missing data reports and reports on impossible values or data discrepancies. CHWs will adjudicate data discrepancies, with research staff holding CHWs accountable for responding promptly, including by contacting study participants if needed. The research team will audit the registers and update the final dataset at the end of the trial.

For Aim 2 fidelity and context outcomes, the research team will administer surveys and conduct qualitative interviews and focus group discussions. These will be audio-recorded, professionally transcribed and translated, and securely stored for analysis. Any personal health identifiers disclosed during the interviews will be removed and names replaced by numbered aliases in the final transcripts. We will store all audio files and de-identified transcripts from qualitative data collection on a secure password-protected network and destroy audio files once transcription is complete. For the Aim 3 costing studies, CHWs will collect time and motion data, and research staff will collect other costing data from facility administrators. We will de-identify all data prior to analysis.

### Statistical power and sample size

Power and sample size calculations for the stepped-wedge, cluster-randomised trial assume two levels of clustering; first, persons with TB nested within health facilities and second, contacts nested within persons with TB. We estimated the intraclass correlation coefficient (ICC) for each level. Drawing on pre-trial empirical data collected at the 12 trial health facilities between 1 January 2022 and 6 March 2022, we conservatively project an average of 11 persons with TB per facility per 8-week block period and an average of one symptomatic contact person per index person with TB. We further assume that 20% of symptomatic contacts in the pre-intervention period and 40% in the post-intervention period will achieve the primary trial outcome of completing TB evaluation. Based on pre-trial data, we estimated an ICC of 0.172 at the facility level and an ICC of 0.647 at the level of index persons with TB. Allowing for two facilities to cross over at each step, six cross-over steps, and 924 symptomatic contacts during the study period, we will have a power of 99% to detect a ≥ 20% increase in the primary outcome. We have estimated this sample size and power using PASS 15 (NCSS Software Kaysville, Utah, USA) and Stata 16 (StataCorp, College Station, Texas, USA) with an extension to account for multiple levels of clustering [43, 44]. Because persons with TB and contacts participate in routine services, we will not limit enrolment if we exceed these numbers, which could be at least four-fold higher if the user-centred implementation strategy is highly effective.

### Statistical methods

The primary analysis will compare the pre- and post-implementation periods, excluding the first eight weeks after transition as a buffer period and the 14 weeks after the announcement of Ebola Virus Disease-related public health restrictions. We will consider the intention-to-treat population only; a per-protocol analysis is not feasible given the site-level nature of the intervention exposure and the lack of granular individual-level fidelity data. Because the primary implementation effectiveness outcome and the secondary clinical effectiveness outcomes are proportions, we will use modified mixed-effects Poisson generalised linear models to determine prevalence risk ratios (PRRs) or proportion ratios (PRs) between the post- and pre-implementation exposure periods. We will use mixed-effects Poisson models for the count outcome of public health effectiveness. For time-to-event outcomes, we will use mixed-effects parametric survival analysis models.

If secular trends are present, we will collapse individual-level data at health facility and cluster levels by trial month. We will estimate the difference in the primary outcome by comparing the pre- and post-implementation periods using aggregated data. We will consider a random-effects logit model, or a random-effects generalized estimating equations (GEE) model, allowing cluster-level aggregated data to vary across time by cluster. The multivariable model will include cluster-level covariates. We will test various correlation structures and select the final model using likelihood-ratio tests and information criteria.

### Subgroup analyses

In the absence of secular trends requiring an aggregated analysis by site and month, additional adjusted analyses will consider outcome differences by age, sex, HIV status, site, and site characteristics, including health centre level, location, and district-level gross-domestic product. We will adjust for trial period as appropriate for stepped-wedge trial analysis. In the presence of secular trends, adjusted analyses will consider age, sex, HIV status, and any other baseline characteristics found to be imbalanced between the pre- and post-implementation periods.

### Oversight and monitoring

The Yale School of Public Health will serve as the study coordinating centre, with Amanda Gupta as the data manager and Noah Kiwanuka and Suzan Nakasendwa as trial statisticians. The Trial Steering Committee includes Mari Armstrong-Hough, Raymond Byaruhanga, Adithya Cattamanchi, David Dowdy, Jessica Haberer, Moorine Sekadde, and Erez Yoeli, and oversees the decisions of the principal investigators, J. Lucian Davis and Achilles Katamba. Given the low-risk behavioural nature of the implementation strategy being evaluated, no data monitoring committee is appointed and no trial audits or interim analyses are planned.

Adverse events occurring after enrolment in contact investigation will be reported to the trial investigators within 24h of initial disclosure and reported to the IRBs within two working days. The IRBs will have the authority to determine if the trial needs to be paused or terminated. No protocol amendments are planned once the trial commences. If an amendment is required based on safety or other indication, we will immediately notify the IRBs. Relevant changes to the study protocol will be communicated to all shareholders as needed after IRB approval.

## Ethics and dissemination

### Research ethics approval

This protocol and the consent forms (Appendices 1, 2, 3, 4 and 5) have been reviewed and approved by the Makerere School of Public Health IRB (#554), the Uganda National Council for Science and Technology (#HS1720ES), and the Yale Human Subjects Committee (#2000023199). These bodies will review the protocol annually and at the time of any protocol modification. All investigators will be given access to a cleaned dataset upon trial completion. Upon publication of the primary outcomes, we will make publicly available a de-identified dataset containing all variables necessary to reproduce our analysis. We will de-identify and store all participant data for up to five years after the study [45]. All methods proposed here will be in accordance with relevant United States and Ugandan guidelines and regulations.

### Informed consent

The Makerere School of Public Health IRB and Yale Human Subjects Committee waived informed consent requirements for Aim 1 because the research poses minimal risk to participants, and obtaining consent is not feasible given the trial’s pragmatic design involving CHWs delivering services in a routine clinic and community settings. The waiver will not adversely affect the rights or welfare of participants because CHWs will request permission before initiating contact investigation, and participants in both arms will receive standard TB contact investigation procedures in both exposure periods. For data collection procedures for Aims 2 and 3, trained research officers will obtain verbal consent (Appendices 1, 2, 3, 4 and 5) from clients and healthcare workers. We will not require re-consent from participants for data use beyond the original aims, if the new analyses pose no greater risk than the original analyses.

### Dissemination plans

We will publish trial results in peer-reviewed journals and disseminate our findings to local communities, policymakers, and the global research community through public and private presentations. We will adhere to the International Committee of Medical Journal Editors guidelines for authorship and ensure equitable recognition of all research team members in publications.

## Trial status

Recruitment of study sites began in April 2021. Randomisation of study sites occurred on 11 February 2022, with participant recruitment beginning on 7 March 2022. Enrolment was interrupted between 17 October 2022 and 15 January 2023 by a public health emergency related to an outbreak of Ebola Virus Disease. We anticipate participant recruitment to end on 27 August 2023.

### Trial registration

The trial registration at ClinicalTrials.gov (NCT05640648) was initiated on 16 November 2022, inadvertently exceeding the 30-day timeframe advised by the International Committee of Medical Journal Editors due to an oversight by the principal investigator. The authors acknowledge that late registration constitutes a methodological limitation. However, the authors note that late registration is unlikely to result in biased reporting in this circumstance, for several reasons. First, this is a protocol manuscript for a not-yet-completed trial, so the trial analysis cannot be changed. Second, the trial is powered to detect the minimum clinically important difference in the trial outcome across all realistic scenarios of enrolment, reducing any incentive to alter the trial design after the start. Third, because the trial is a complete stepped-wedge, cluster-randomized implementation trial, its sample size is fixed by the duration of the enrolment period and cannot be changed after the trial starts. Last, this is a trial of a social and behavioural intervention, reducing any financial incentive to delay registration.

## Discussion

The three pillars of the WHO END TB strategy are Integrated, Patient-Centred Care and Prevention, Bold Strategies and Supportive Systems, and Intensified Research and Innovation. This pragmatic, stepped-wedge cluster-randomised implementation trial leverages all three of these pillars, including novel and interdisciplinary implementation research methods; community-engaged co-design method to tailor delivery strategies that support community members and healthcare workers; and integrated person-centred TB diagnostic, treatment, and prevention services.

This ongoing trial has several strengths that enhance its novelty and rigor. First, the tailored, multi-component implementation strategy was developed using human-centred design and targets both client and healthcare-worker barriers to uptake and completion of TB contact investigation [46]. Second, the trial employs a pragmatic study design to enhance generalizability, from enrolment criteria that ensure that the enrolled population is broadly representative; flexibility and adaptability with respect to delivery of the implementation strategy and selection of a relevant comparator strategy; and the assessment of outcomes relevant to people, providers, and policymakers [47, 48]. Third, the waitlisted, cluster-randomised trial design reduces the risk of cross-contamination between interventions and enables multi-level assessments of factors influencing implementation fidelity.

The trial also has some potential limitations. The stepped wedge trial design increases the study’s vulnerability to secular trends, such as enrolment and recruitment interruptions related to a recent Ebola Virus Disease outbreak in Uganda. In addition, this trial is vulnerable to several additional challenges for power estimation and analysis common to stepped-wedge trials conducted in real-world settings, including small and imbalanced cluster-sizes; clustering at multiple levels; and rare outcomes, especially in usual care periods [49]. A key objective of this study protocol is to pre-specify our approach to these challenges to enhance rigor and reproducibility in reporting trial outcomes.

In conclusion, the proposed pragmatic, quasi-experimental implementation trial will inform efforts to find and prevent undiagnosed persons with TB in high-burden setting using contact investigation. It will also help assess the suitability of human-centred design and communities of practice for tailoring strategies to implement and sustain evidence-based interventions in Uganda and other low-and-middle-income countries.

### Supplementary Information

**Additional file 1.**

**Additional file 2.**

## Data Availability

Upon publication of the primary outcomes, we will make publicly available a de-identified dataset (accessible through J. Lucian Davis, lucian.davis@yale.edu) containing all variables necessary to reproduce our analysis.

## Abbreviations

CHR: Community Health Rider
CHW: Community Health Worker
TB: Tuberculosis
TPT: Tuberculosis Preventative Therapy
WHO: World Health Organization
