# Supplementary material for: A user-centred implementation strategy for tuberculosis contact investigation in Uganda: protocol for a stepped-wedge, cluster-randomised trial
Source: BMC Public Health. 2023 Aug 17;23:1568. doi: 10.1186/s12889-023-16510-0 (PMC10436440; doi:10.1186/s12889-023-16510-0)
Supplement: Supplementary file 1 — Additional file 1. [file 12889_2023_16510_MOESM1_ESM.docx]

**APPENDICES – Consent Documents**

**Appendix 1.** Verbal Consent for Qualitative Data Collection from TB Patients and Close Contacts

**MAKERERE UNIVERSITY COLLEGE OF HEALTH SCIENCES**

**YALE UNIVERSITY**

**VERBAL CONSENT TO PARTICIPATE IN A RESEARCH STUDY**

Hello, my name is __________ from Makerere University. I’d like to ask you to participate in a research study about TB contact investigation. We are asking you to be in this study because you were visited by a community health worker to offer TB screening to you or your close contacts at the household. We are interested in your opinions on this subject.

If you agree to be in this study, we will ask you to participate in a focus group discussion and/or in-depth interview and/or survey to explore your opinions about TB contact investigation. The discussion will take about one hour. It will be audio-recorded, transcribed and de-identified, and then destroyed.

Your answers to questions in the study will be kept confidential and only key research staff will listen to the recordings. Taking part in this study is optional, and you can tell me if you want to stop being in the study at any time. Refusing to participate or ending your participation will not have any effect on your healthcare. Participating in this interview is voluntary. You will not be charged any costs for participating, and you will receive 20,000 Uganda shillings to compensate you for your time.

Do you have any questions about the study?

Would you like to participate?

If you have questions about this study in the future, you can contact the study coordinator at phone number ____________.

**Appendix 2.** Verbal consent for Qualitative Data Collection from Health Workers

**MAKERERE UNIVERSITY COLLEGE OF HEALTH SCIENCES**

**YALE UNIVERSITY**

**VERBAL CONSENT TO PARTICIPATE IN A RESEARCH STUDY**

Hello, my name is __________ from Makerere University. I’d like to ask you to participate in a research study about TB contact investigation. This is an important intervention for finding undiagnosed people with TB in the community. We are asking you to be in this study because we are interested in your opinions of and/or experiences with strategies designed to improve the delivery of TB contact investigation and/or other health services.

If you agree to be in this study, we will ask you to participate in a focus group discussion and/or in-depth interview and/or survey, in order to explore your opinions about the new TB contact investigation strategy. Each activity will take about 30-60 minutes. If performed, interviews and focus groups will be audio-recorded, transcribed, and de-identified, and then destroyed.

Your answers to questions in the study will be kept confidential. Only key research staff will listen to the recordings or view your individual survey responses. Taking part in this study is optional, and you can tell me if you want to stop being in the study at any time. Refusing to participate or ending your participation will not have any effect on your employment and any responses will be solely used for research purposes and not for performance evaluation. Participating in this interview is voluntary. You will not be charged for participating. You will receive 20,000 Uganda shillings to compensate you for your time.

Do you have any questions about the study?

Would you like to participate?

If you have questions about this study in the future, you can contact the study coordinator at phone number ____________.

**Appendix 3.** Verbal Consent to Record Health Workers during Community of Practice Meetings

**MAKERERE UNIVERSITY COLLEGE OF HEALTH SCIENCES**

**YALE UNIVERSITY**

**VERBAL CONSENT TO PARTICIPATE IN A RESEARCH STUDY**

Hello, my name is __________ from Makerere University. I’d like to ask you to participate in a research study about ways in which we can improve TB contact investigation. This is an important intervention for finding undiagnosed people with TB in the community. We are asking you to be in this study because you will participate in quality improvement meeting called a community of practice that will be taking place at your clinic. We are interested recording the meeting proceedings to gain insight on whether or not the meeting was an implementation success.

If you agree to be in this study, every time you participate in these meetings we shall record all meeting proceedings using a recorder at your facility and these will be transferred to a secure password protected folder Box Secure. Recordings will NOT be transcribed or translated. All recordings will be deleted within 5 years, following completion of the COP fidelity checklist.

All opinions shared during these meetings in this will be kept confidential. Only key research staff will listen to the recordings. Taking part in this study is optional, and you can tell me if you want to stop being in the study at any time. Refusing to participate or ending your participation will not have any effect on your employment and any responses will be solely used for research purposes and not for performance evaluation.

Do you have any questions about the study?

Would you like to participate?

If you have questions about this study in the future, you can contact the study coordinator at phone number ____________.

**Appendix 4.** Verbal Consent for Time and Motion Studies from TB Patients and Close Contacts

**MAKERERE UNIVERSITY COLLEGE OF HEALTH SCIENCES**

**YALE UNIVERSITY**

**VERBAL CONSENT TO PARTICIPATE IN A RESEARCH STUDY**

Hello, my name is __________, a researcher from Makerere University. I’d like to ask you to participate in a research study about home-based TB contact investigation. We are asking you to be part of the economic evaluations of this study because you were visited by a community health worker to offer TB screening to you or your close contacts at the household. We want to understand the cost involved in participating in such a public health program from the perspective of the user.

If you agree to be in this study, one of our staff will follow a Community Health Worker as they conduct Contact Investigation activities on you and later go through a questionnaire assessing the costs and missed salary to you for participating in such a public health program.

Please note that this survey is not conducted to evaluate your response and the data will only be used for research purposes. Your recorded activities will be kept confidential. Therefore, you should not change your behavior in any way as a result of this survey.

Participating in this activity is voluntary. If you have any concerns or questions about this survey, we can take down your phone number and have the project investigator contact you or give you the phone number of the Research Ethics Committee at Makerere University School of Public Health.

Do you have any questions about the study?

Would you like to participate?

If you have questions about this study in the future, you can contact the study coordinator at phone number ____________.

**Appendix 5.** Verbal Consent for Time and Motion Studies from Health Workers

**MAKERERE UNIVERSITY COLLEGE OF HEALTH SCIENCES**

**YALE UNIVERSITY**

**VERBAL CONSENT TO PARTICIPATE IN A RESEARCH STUDY**

Hello, my name is __________, a researcher from Makerere University. I’d like to ask you to participate in a research study about the time and costs for TB contact investigation. This is an important intervention for finding undiagnosed people with TB in the community. As part of the research study, the clinic where you work has been or will soon be implementing an enhanced TB contact investigation intervention strategy *(Tuli Wamu Nawe)*. This intervention is a special type of TB contact investigation that focuses on the specific needs and concerns of each TB contact and uses Community Health riders (CHRs) for transportation. We are trying to compare these two types of TB contact investigation for our research to see how much each will cost.

If you agree to be in this study, we would kindly ask to track your activities related to TB contact Investigation for approximately one full week using a survey form we made. We would ask you to do this at two separate times: once during the period where you use standard TB contact investigations and again after you begin an enhanced implementation strategy (*Tuli Wamu* Nawe). We will provide you an initial training, lasting approximately 10 minutes, to show you what sorts of information will be recorded.

Please note that this survey is not conducted to evaluate your performance and the data will only be used for research purposes. Your recorded activities will be kept confidential. Therefore, you should not change your behavior in any way as a result of this survey.

Participating in this activity is voluntary. You will not be charged any costs for participating. If you have any concerns or questions about this survey, we can take down your phone number and have the project investigator contact you, or give you the phone number of the Research Ethics Committee at Makerere University School of Public Health.

Do you have any questions about the study?

Would you like to participate?

If you have questions about this study in the future, you can contact the study coordinator at phone number ______________________.
